# Efficacy and safety of a biomarker-driven cetuximab-based treatment regimen over 3 treatment lines in mCRC patients with *RAS/BRAF* wild type tumors at start of first line: The CAPRI 2 GOIM trial

**DOI:** 10.3389/fonc.2023.1069370

**Published:** 2023-02-13

**Authors:** Giulia Martini, Davide Ciardiello, Stefania Napolitano, Erika Martinelli, Teresa Troiani, Tiziana Pia Latiano, Antonio Avallone, Nicola Normanno, Massimo Di Maio, Evaristo Maiello, Fortunato Ciardiello

**Affiliations:** ^1^ Dipartimento di Medicina di Precisione, Oncologia Medica, Università degli Studi della Campania “Luigi Vanvitelli”, Napoli, Italy; ^2^ Oncologia Medica, Fondazione Istituto di Ricovero e Cura a Carattere Scientifico (IRCCS) Casa Sollievo della Sofferenza, San Giovanni Rotondo, Italy; ^3^ Istituto Nazionale per lo Studio e la Cura dei Tumori “Fondazione Giovanni Pascale”– Istituto di Ricovero e Cura a Carattere Scientifico (IRCCS), Oncologia Clinica Sperimentale Addome, Napoli, Italy; ^4^ Biologia Cellulare e Bioterapie, Istituto Nazionale per lo Studio e la Cura dei Tumori “Fondazione Giovanni Pascale”–Istituto di Ricovero e Cura a Carattere Scientifico, Napoli, Italy; ^5^ Dipartimento di Oncologia, Università di Torino, Azienda Ospedaliera Mauriziana, Torino, Italy

**Keywords:** colorectal cancer, EGFR, liquid biopsy, biomarker, cetuximab

## Abstract

**Background:**

Monoclonal antibodies targeting EGFR such as cetuximab or panitumumab represent a major step forward in the treatment of *RAS* wild type (WT) metastatic colorectal cancer (mCRC). Unfortunately, primary and acquired resistance mechanisms occur, with a huge percentage of patients succumbing to the disease. In the last years, *RAS* mutation has been identified as the main molecular driver that determine resistance to anti-EGFR monoclonal antibodies. Liquid biopsy analysis allows to a dynamic and longitudinal assessment of mutational status during mCRC disease and has provided important information on the use of anti-EGFR drugs beyond progression or as rechallenge strategy in patients with *RAS* WT tumors.

**Methods:**

The phase II CAPRI 2 GOIM trial investigates the efficacy and safety of a bio-marker-driven cetuximab-based treatment regimen over 3 treatment lines in mCRC patients with *RAS/BRAF* WT tumors at start of first line.

**Discussion:**

The aim of the study is to identify patients with *RAS/BRAF* WT tumors defined as “addicted” to an-anti EGFR based treatment along three lines of therapy. Moreover, the trial will evaluate the activity of cetuximab re-introduction in combination with irinotecan as 3^rd^ line therapy as rechallenge for patients that will be treated in second line with FOLFOX plus bevacizumab, having a *RAS/BRAF* mutant disease at progression after FOLFIRI plus cetuximab first line. A novel characteristic of this program is that the therapeutic algorithm will be defined at each treatment decision (*first line, second line and third line*) in a prospective fashion in each patient by a liquid biopsy assessment of *RAS/BRAF* status by a comprehensive 324 genes Foundation One Liquid assay (Foundation/Roche).

**Trial registration:**

EudraCT Number: 2020-003008-15, ClinicalTrials.gov identifier: NCT05312398.

## Introduction

### Metastatic colorectal cancer

Colorectal cancer (CRC) is one of the most diagnosed cancers worldwide, with 1.8 million new cases per year (1). In the last years, the use of standard chemotherapy and targeted agents has considerably increased the prognosis of metastatic colorectal cancer (mCRC) patients, with an improvement in median overall survival (OS) to approximately 36 months (2). Several clinical trials have explored the use of cetuximab or panitumumab monoclonal antibodies (mAbs) to target the Epidermal Growth Factor Receptor (EGFR) in the treatment of *RAS* wild type (WT) metastatic colorectal cancer (mCRC) among different lines of treatment (3). However, despite the huge improvement of patient responses, the response is impaired due to the presence of innate or acquired mechanisms of resistance to anti-EGFR blockade (4). In the past years, several molecular biomarkers have been identified in retrospective preclinical and clinical analyses to predict resistance to cetuximab and panitumumab. Among these, *RAS* mutational status is today the principal biomarker of poor response to an anti-EGFR drugs and patients with *RAS*-mutated mCRC are excluded by their treatment (5). In addition, other components of the EGFR signalling pathway determine intrinsic or acquired resistance to EGFR inhibitor including mutation of *BRAF* and *PI3KCA*; amplification of *HER2*, *MET* and *KRAS*; and loss of *PTEN* expression (6, 7). All these alterations seem converge to the MAPK-ERK intracellular driver, which is over-activated and is responsible of tumor survival even when EGFR inhibitors are used (8). The molecular scenario is complicated by the presence of inter-tumor and intratumor heterogeneity of resistance mechanisms, with different molecular clones present at the same time in a patient and even in the same organ (9). In recent years, we have assisted to a widespread use of circulating tumor DNA (ctDNA) testing over the tissue biopsy for the detection in blood of mutations that characterize resistance to target therapy in mCRC (10, 11). Morelli et al. have previously demonstrated how *RAS* and *EGFR* mutant alleles exponentially decline when treatment with EGFR inhibitors is interrupted, with an half-life of nearly 4 months (12, 13). These data provide strong support for the so called rechallenge strategy with anti-EGFR monoclonal antibodies, in a subset of patients treated in front line with chemotherapy plus cetuximab or panitumumab followed by an EGFR free interval of at least 4 months after progression. Different trials are underway, to prospectively study rechallenge treatment with cetuximab and panitumumab. Phase II clinical trials have been published to date as the CAVE mCRC trial, in which rechallenge strategy in refractory patients with *RAS* WT mCRC with cetuximab plus avelumab, an anti-programmed death ligand 1 (PD-L1) monoclonal antibody has demonstrated clinical evidence of improved overall survival, with the highest benefit obtained in those patients with baseline *RAS/BRAF/EGFR* WT circulating tumor DNA (ctDNA) (14).

### Rationale of the study

The rationale of anti-EGFR treatment beyond progression of disease comes from our previous CAPRI GOIM Study, performed in 25 Italian centres in which 340 patients with *KRAS* exon 2 WT mCRC received a first line treatment with FOLFIRI plus cetuximab; of these, 153 mCRC patients, at progression after responding to FOLFIRI plus cetuximab, were treated with FOLFOX or with FOLFOX plus cetuximab in a randomized phase II study (15). In addition, the CAPRI GOIM clinical program has performed extensive translational research with the establishment of a selected 22 multigene next generation sequencing (NGS) test for DNA extracted from tumor tissue and of a selected RAS gene. Beaming technology has been used for circulating free tumor DNA extracted from plasma. The main findings of the CAPRI GOIM clinical research project can be summarized as follows: FOLFIRI plus cetuximab is an effective front line treatment in molecularly selected patients with mCRC. Efficacy is similar in fit elderly patients. Efficacy is higher in patients with *KRAS/NRAS/BRAF/PIK3CA* WT tumors. RAS testing by liquid biopsy is feasible and predicts efficacy of FOLFIRI plus cetuximab. Second line FOLFOX plus cetuximab is a promising therapeutic approach in patients with *KRAS/NRAS/BRAF/PIK3CA* WT tumors that benefited from first line FOLFIRI plus cetuximab (16). Extended and comprehensive multigene assessment by NGS allows the identification of potential rare gene alterations that could be responsible for resistance to cetuximab in *KRAS/NRAS/BRAF/PIK3CA* WT tumors.

Based on the findings of the CAPRI GOIM trial, the CAPRI 2 GOIM trial has the purpose of investigate the efficacy and safety of a biomarker-driven cetuximab-based treatment regimen over 3 treatment lines in mCRC patients with *RAS/BRAF* WT tumors at start of first line.

**Figure 1:**
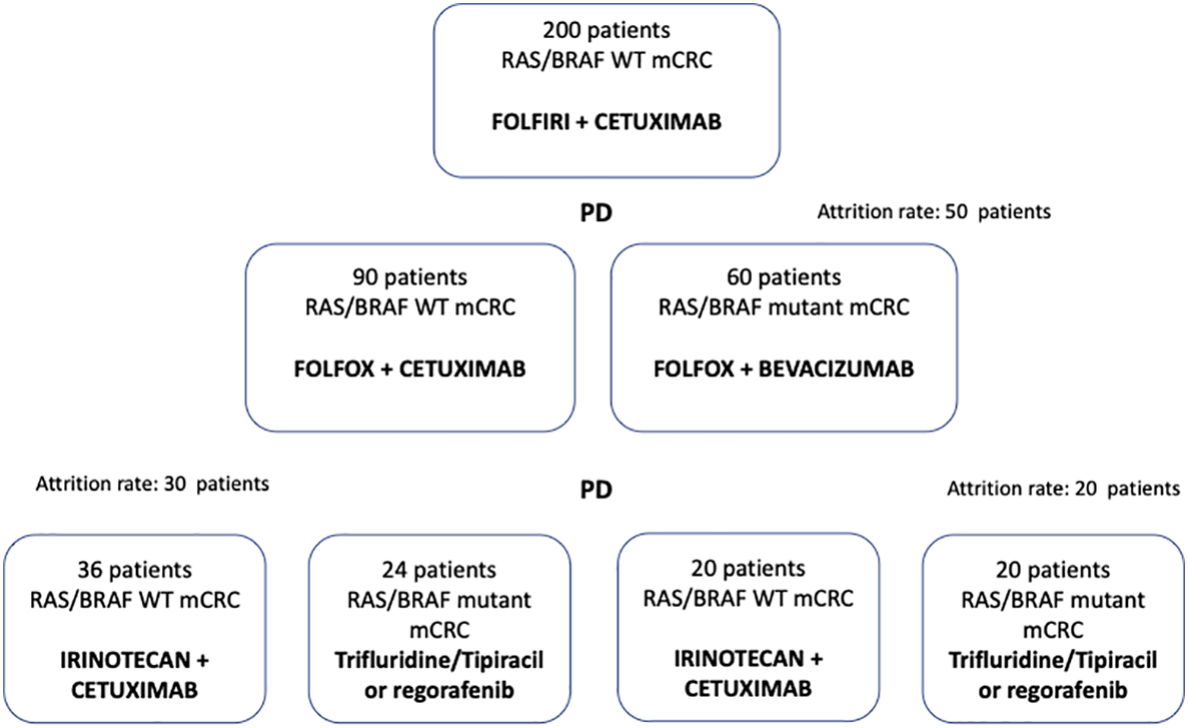
Capri 2 GOIM clinical trial consort diagram.

In the present paper we describe and discuss the scientific and clinical rationale, the design and treatment lines of the CAPRI 2 GOIM clinical trial (**Figure 1**).

## Materials and methods

**Figure 2:**
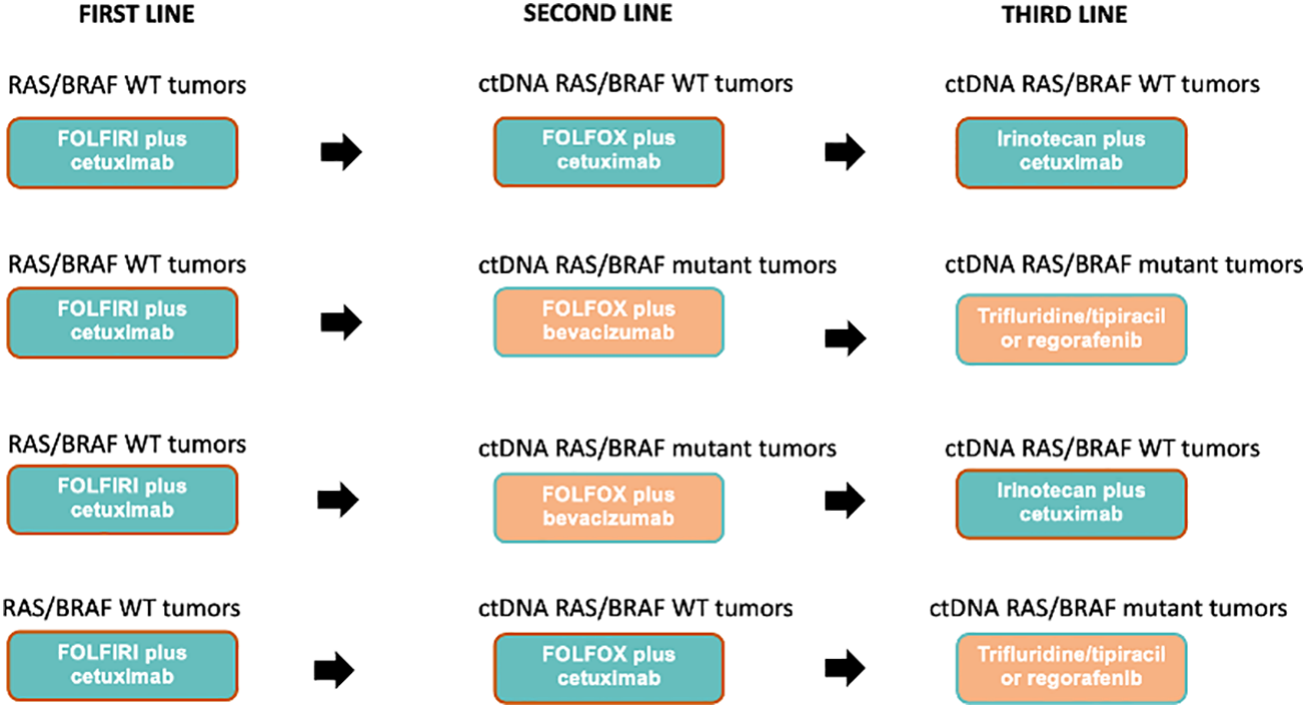
Possible treatment sequences based on ctDNA plasma analysis.

Based on dynamic and longitudinal liquid biopsy assessment of RAS/BRAF status, that will be prospectively performed before each line of treatment, 200 mCRC patients will be treated with cetuximab in combination with chemotherapy throughout three lines of therapy, as follows: FOLFIRI plus cetuximab (first line); FOLFOX plus cetuximab (second line); irinotecan plus cetuximab (third line) in case of RAS/BRAF WT at each time point of progression. If after the first line progression, the liquid biopsy assessment indicates *RAS* and or *BRAF* mutant status, patients will receive FOLFOX plus bevacizumab as the second line of therapy. If after the second line progression, the liquid biopsy assessment indicates *RAS* and or *BRAF* mutant status, patients will be treated with regorafenib or trifluridine-tipiracil (investigator’s choice), as third line of therapy (**Figure 1**). Each treatment will be administered using standard doses and schedules until progression of disease or unacceptable toxicity (**Figure 2**).

### Technical procedures to manage diagnostic samples from enrolled patients:

Liquid biopsy: Two blood samples will be obtained before each line of treatment (total of 29 mL): one will be shipped to Foundation Roche Germany for extended RAS/BRAF molecular analysis, the second will be processed for additional translational analyses. Liquid biopsy assessment will be performed with a comprehensive 324 genes Foundation One Liquid NGS assay (Foundation/Roche). Briefly, blood samples will be collected before each line of treatment and will be shipped to Foundation Roche Germany for extended RAS/BRAF molecular analysis. Circulating cell-free DNA will be isolated from plasma and analyzed with the Foundation One Liquid assay (Foundation/Roche). This assay assesses SNVs, indels, CNVs and fusions in 324 cancer related genes (https://www.foundationmedicine.com/genomic-testing/foundation-one-liquid).

Tissue analysis: Baseline Formalin-fixed-paraffin-embedded (FFPE) of primary tumor or metastasis will be analyzed by local laboratory for the determination of RAS/BRAF mutational status.

Moreover, a baseline FFPE sample will be shipped to Foundation Roche Germany for molecular analysis. FFPE samples will be analyzed with the Foundation One CDx assay (Foundation/Roche), which covers single nucleotide variants (SNVs), indels, copy number variations (CNV) and fusions in 324 cancer- related genes (https://www.foundationmedicine.com). An additional baseline FFPE sample will be shipped to the Cell Biology and Biotherapy Unit, Istituto Nazionale Tumori “Fondazione Giovanni Pascale” IRCCS, Napoli for further translational analyses.

Translational analyses: additional 12 mL of whole blood will be collected. Plasma and peripheral blood mononuclear cells (PBMC) will be collected, stored at -70/-80 °C (preferred) until shipment on dry ice to the Cell Biology and Biotherapy Unit, Istituto Nazionale Tumori “Fondazione Giovanni Pascale” IRCCS, Napoli.

### Patients

Patients eligible for inclusion in the CAPRI 2 GOIM trial have to meet all of the following criteria at the start of first line treatment: - Histologically proven diagnosis of colorectal adenocarcinoma - Diagnosis of metastatic disease - *RAS* and *BRAF* WT status of FFPE analysis of primary CRC and/or distant metastasis - Measurable disease.

Other eligible criteria are described in **Tables 1**, **2**.

**Table 1:** Patients eligible for inclusion in this study have to meet all of the following criteria at the start of first line treatment.

1. Histologically proven diagnosis of colorectal adenocarcinoma
2. Diagnosis of metastatic disease
3. RAS and BRAF wild-type status of FFPE analysis of primary colorectal cancer and/or related metastasis
4. Measurable disease according to Response Evaluation Criteria in Solid Tumors (RECIST criteria, vers.1.1)
5. Male or female patients ≥ 18 years of age
6. ECOG Performance Status 0,1
7. Adequate bone marrow, liver and renal function assessed within 14 days before starting study treatment as defined by the following parameters: Bone marrow: Absolute Neutrophil Count (ANC) ≥ 1.5 x 10^9^/L Hemoglobin (Hgb) ≥ 9 g/dL Platelets ≥ 100 x 10^9^/L Liver function: Serum total bilirubin ≤ 1.5 x upper limit of normal (ULN) Aspartate aminotransferase (AST) (serum glutamic oxaloacetic transaminase [SGOT]) and ALT (SGPT) ≤ 2.5 x ULN, except in patients with tumor involvement of the liver who must have AST and ALT ≤ 5 x ULN Renal function: Serum creatinine ≤ 1.5 x ULN or 24-hour clearance ≥ 50 mL/min
8. If female and of childbearing potential, have a negative result on a pregnancy test performed a maximum of 7 days before initiation of study treatment
9. If female and of childbearing potential, or if male, agreement to use adequate contraception (e.g., abstinence, intrauterine device, oral contraceptive, or double-barrier method), during the study and until at least 3 months after last dose of study treatment administration, based on the judgment of the Investigator or a designated associate
10 Signed informed consent obtained before screening.

**Table 2:** Patients eligible for this study must not meet any of the following criteria at the start of first line treatment.

11.Any contraindication to the use of cetuximab, Irinotecan, 5-FU, oxaliplatin, folinic acid, bevacizumab, trifluridine-tipiracil, regorafenib
12. Active uncontrolled infections, active disseminated intravascular coagulation or history of interstitial lung disease
13. Past or current history of malignancies other than colorectal carcinoma, except for curatively treated basal and squamous cell carcinoma of the skin cancer or *in situ* carcinoma of the cervix
14. Pregnancy (exclusion to be ascertained by a beta hCG test
15. Breastfeeding
16. Fertile women (<2 years after last menstruation) and men of childbearing potential not willing to use effective means of contraception•
17. Myocardial infarction, unstable angina pectoris, balloon angioplasty (PTCA) with or without stenting within the past 12 months before inclusion in the study, Grade III or IV heart failure (NYHA classification)
18. Cardiac arrhythmias requiring anti-arrhythmic therapy, with the exception of beta blockers or digoxin
19. Medical or psychological impairments associated with restricted ability to give consent or not allowing conduct of the study
20. Previous chemotherapy for the colorectal cancer with the exception of adjuvant treatment, completed at least 6 months before entering the study
21. Participation in a clinical study or experimental drug treatment within 30 days prior to study inclusion or during participation in the study
22. Known or clinically suspected brain metastases
23. History of acute or subacute intestinal occlusion or chronic inflammatory bowel disease or chronic diarrhoea
24. Severe, non-healing wounds, ulcers or bone fractures
25. Uncontrolled hypertension
26. Marked proteinuria (nephrotic syndrome)
27. Known DPD deficiency (specific screening not required)
28. Known history of alcohol or drug abuse
29. A significant concomitant disease which, in the investigating physician’s opinion, rules out the patient’s participation in the study
30. Absent or restricted legal capacity

## Study endpoints

### Primary endpoint

The primary endpoint of the study is the Response rate (RR) for each line of treatment according to Response Evaluation Criteria in Solid Tumors (RECIST) v1.1.

### Secondary endpoints

- Progression Free Survival (PFS): measured from the start of therapy until the first observation of disease progression or death due to any cause.
- Overall Survival (OS): calculated from the start of the study treatment until death.
- Safety: Adverse events graded according to NCI CTCAE v 5.0.
- Molecular profiles of tumor tissue and liquid biopsy: molecular analysis of formalin fixed paraffin embedded (FFPE) tumor tissue, which is representative of the primary tumor or of a metastatic site at the diagnosis of mCRC, will be performed before the first line, whilst blood samples for liquid biopsy will be collected before each line of treatment.

### Exploratory endpoints

An additional aliquot of the blood/plasma/fecal samples will be stored for further translational studies. The analysis on tissue and plasma samples will be performed by Foundation Roche laboratories in Germany using a comprehensive 324 genes Foundation One Liquid assay (Foundation/Roche).

The analysis of fecal samples for gut microbioma study will be performed by the gastroenterology unit, Casa Sollievo della Sofferenza, *Via* padre Pio 7d 70013, San Giovanni Rotondo (FG).

## Statistical analysis

The primary analysis of response will be performed in a modified intention-to-treat population (mITT), defined as all enrolled patients with RAS/BRAF wild-type tumors who received at least one dose of study treatment. No reliable prospective data for defining the percentage of WT and mutated patients according to liquid biopsy after FOLFIRI plus Cetuximab as first line and any second line FOLFOX plus bevacizumab are available at the time of the trial design. However, we assume that acquired RAS or BRAF mutations detectable in the plasma occur as often as 40% at the beginning of the second line. Moreover, at the beginning of the third line we assume that 40% of patients who received a continuous EGFR inhibition in second line will acquire a mutation in RAS or BRAF genes; on the other hand, in about half of patients who received FOLFOX plus bevacizumab as second line, RAS or BRAF WT status will be restored. On the basis of this assumption, we calculated that 200 patients will receive a first line with FOLFIRI plus cetuximab. In addition, FOLFOX plus cetuximab as second line for patients WT on liquid biopsy would provide the trial with a power of 80% to detect a significantly higher response rate of 35% compared to historical control of 20%. P0 = 0.20 Pt = 0.35; the study requires a sample size of 56, achieves 81% power to detect a difference (P1-P0) of 0.15 using a one-sided binomial test. The target significance level is 0.05. The actual significance level achieved by this test is 0.0432. These results assume that the population proportion under the null hypothesis is 0.2000. If the number of responses is 12 or less, the null hypothesis that P <= 0.20 is accepted with a target error rate of 0.19 (1-power=1-0.81). The expected response rate increase in the third line will be as follow: P0 = 0.02 Pt = 0.15. The study requires a sample size of 28; achieves 81% power to detect a difference (P1-P0) of 0.13 using a one- sided binomial test. The target significance level is 0.025. The actual significance level achieved by this test is 0.018. These results assume that the population proportion under the null hypothesis is 0.02. If the number of responses is 8 or less, the hypothesis that P <= 0.02 is accepted with a target error rate of 0.19. We will determine PFS and OS using the Kaplan-Meier method and the median survival estimate of the OS and PFS and the related confidence interval (CI) will be compared to the lower bound of the CI observed in the historical control. This comparison will be made only for descriptive purposes.

## Discussion

The CAPRI 2 GOIM clinical trial is the first trial to explore the use of anti-EGFR treatment for three subsequent treatment lines in those patients defined as addicted to anti-EGFR blockade. Moreover, the study will also evaluate the activity and efficacy of cetuximab plus irinotecan rechallenge for those patients treated in second line with chemotherapy plus anti-angiogenic drugs (FOLFOX plus bevacizumab), having a *RAS* or *BRAF* mutant disease at the time of progression after FOLFIRI plus cetuximab first line treatment. Although exclusion criteria of the CAPRI 2 GOIM clinical trial do not refer to patients with microsatellite instable (MSI) tumors, these should be treated with pembrolizumab in first line setting, as international guidelines recommend according to the results of KEYNOTE-177 trial (17). Therefore, these patients are not the right candidates for CAPRI 2 GOIM trial, for which the principal objective is to investigate how three lines of EGFR-based treatment could be effective in patients with RAS/BRAF WT tumors. Moreover, at the time of trial initiation in Italy pembrolizumab was not yet reimbursed for first line treatment in patients with MSI mCRC. For this reason, if a patient enrolled in the trial shows microsatellite instability at the molecular analysis, investigators should discuss with the patient the best treatment option, as in this case pembrolizumab, and therefore evaluate to exit from the trial.

Liquid biopsy assessment with a comprehensive 324 genes Foundation One Liquid NGS assay (Foundation/Roche) will provide not only an integrated analysis of *RAS* and *BRAF* genes mutational status along all the duration of the trial, but also an extensive study of potential biomarkers of response to cetuximab based treatment that, together with the analysis of the influence of gut microbiome on anti-tumor activity will allow to a better tailored anti-cancer treatment in mCRC.

The CAPRI 2 GOIM clinical trial will enroll 200 patients from 25 italian centers. The first patient has been enrolled in July 2021. We estimate the end of enrollment in September 2023 and the end of the study on July 2026.

## Author contributions

GM, DC, EvM, FC: clinical trial and protocol development. GM, SN, DC, EM, and TT have written the manuscript for the study protocol with the support of MM, NN and AA. All authors contributed to the article and approved the submitted version.
